# Engineering the Production of Major Catechins by *Escherichia coli* Carrying Metabolite Genes of *Camellia sinensis*

**DOI:** 10.1100/2012/529031

**Published:** 2012-05-01

**Authors:** Kabir Mustapha Umar, S. M. Abdulkarim, Son Radu, Azizah Abdul Hamid, Nazamid Saari

**Affiliations:** Department of Food Science, Faculty of Food Science and Technology, Universiti Putra Malaysia UPM 43400, Serdang Selangor, Malaysia

## Abstract

A mimicked biosynthetic pathway of catechin metabolite genes from *C. sinensis*, consisting of flavanone 3 hydroxylase (F3H), dihydroflavonol reductase (DFR), and leucoanthocyanidin reductase (LCR), was designed and arranged in two sets of constructs: (a) single promoter in front of F3H and ribosome-binding sequences both in front of DFR and LCR; (b) three different promoters with each in the front of the three genes and ribosome-binding sequences at appropriate positions. Recombinant *E. coli* BL (DE3) harbouring the constructs were cultivated for 65 h at 26°C in M9 medium consisting of 40 g/L glucose, 1 mM IPTG, and 3 mM eriodictyol. Compounds produced were extracted in ethyl acetate in alkaline conditions after 1 h at room temperature and identified by HPLC. Two of the four major catechins, namely, (−)-epicatechin (0.01 ) and (−)-epicatechin gallate (0.36 mg/L), and two other types ((+)-catechin hydrate (0.13 mg/L) and (−)-catechin gallate (0.04 mg/L)) were successfully produced.

## 1. Introduction

**Figure 1:**
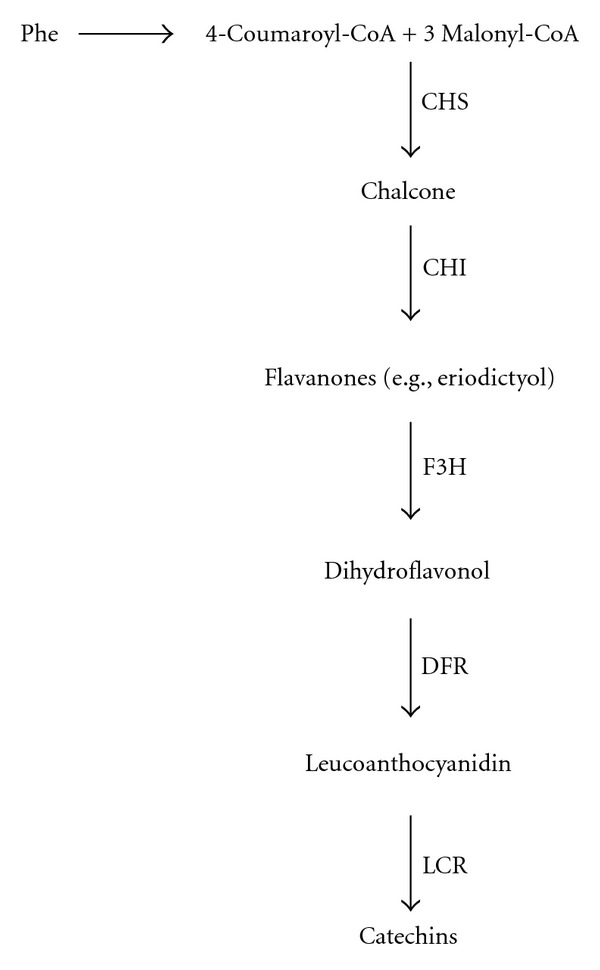
The proposed pathways for the biosynthesis of catechins in tea leaves. Enzymes are CHS: chalcone synthase; CHI: chalcone isomerase; F3H: flavanone 3-hydroxylase; DFR: dihydroflavonol reductase; LCR: leucoanthocyanidin reductase. (Adopted from Park et al. [1]).

Catechins are synthesized from phenylalanine, an aromatic amino acid (AAA) derived from the shikimate pathway, [2]. Phenylalanine, a derivative of the shikimate pathway is the first metabolic node of the phenylpropanoid pathway. The pathway is a metabolic tree with many branches in which a plethora of phenolic compounds including flavonoids are synthesized. The study of Park et al. [1] on subtractive cDNA library and EST database in *Camellia sinensis* (tea plant) showed a high- level expression of primarily three genes, namely, *F3H*, *DFR*, and *LCR* encoding flavanone 3-hydroxylase, dihydroflavonol 4-reductase, and leucoanthocyanidin reductase, in young leaves than in mature leaves. The finding further proposes a biosynthetic pathway of catechins. Chalcone synthase (CHSs) condenses one molecule of 4-coumaryol-CoA and three molecules of malonyl-CoA to produce a chalcone, which is later converted into dihydroflavonol through a stereospecific hydroxylation by F3H. Dihydroflavonol is then stereospecifically reduced by DFR to result in leucoanthocyanin that is finally used as a substrate for the production of catechins by the action of LCR (Figure 1) [3]. Despite this extensive progress, attempts have not been made to mimic the pathway to prove the production of catechins in other hosts.

It is important to note that catechins have drawn a lot of attention due to the variety of their properties. Among the most studied properties are antioxidative [4], antiinflammatory [5], antiobesity [6], and antidiabetic [7], just to mention a few.

The aforementioned properties of catechins have made them potential functional ingredients in food stuff and feed stuff that improve the quality of food products with increased shelf life [8]. These potentials have led to the development of food products like tea beverages, cereal bars, as well as ice creams, confectionaries and pet food that contain tea as an active ingredient [9]. A number of methods have been industrially developed by investigators to maximize the content of catechins in food and drinks [10, 11], but the sources of these substances have solely been from tea leaves. One major drawback of these sources is its total reliance on agriculture, which may result in shortages due to unfavourable environmental conditions caused by the ever changing climates.

It is worth highlighting that metabolic engineering strategies have recently been developed for the standardized biosynthesis of flavonoids in the recombinant *Saccharomyces cerevisiae* and *Escherichia coli* [12–16] by incorporating genes in the biosynthetic pathway. To the researchers' best knowledge, none of the results have produced any of the four primary catechins, but the potential for applying metabolic engineering strategies exists for the synthesis of other natural and nonnatural flavonoids. Thus, the production of pure catechins by *E. coli* strains carrying a cluster of F3H, DFR and LCR from *C. sinensis *and cultivated in the presence of eriodictyol, a flavanone, is presented in the current study.

## 2. Materials

### 2.1. Source and Handling of *C. sinensis* Leaves

Leaves were collected from BOH Tea Plantation which is located in Cameron Highlands, in Malaysia. The leaves were then brought to the laboratory in clean polythene bags for immediate analysis or otherwise stored in a freezer at −20°C for future use.

### 2.2. Strains, Plasmids, and Chemicals

*E. coli* strains (JM109 and BL21 (DE3)) were purchased from Fermentas (Maryland, USA) and plasmids pET26b, pET25b, and pUC18 were purchased from Novagen (Merck KGaA, Darmstadt, Germany). Ampicillin (50 *μ*g/mL) and Kanamycin (30 *μ*g/mL and 5 *μ*g/mL) were used when necessary. X-gal and IPTG were purchased from Nacalai Tesque (Kyoto, Japan) and Fermentas, respectively. Eriodictyol was purchased from Sigma (St. Louis, MO, USA). The restriction enzymes, DNA ligation kit, genomic DNA purification kit, GeneJET plasmid Miniprep kit, bacterial transformation kit, and IPTG were purchased from Fermentas, whereas KOD Hot Start DNA polymerase was purchased from Novagen. All other Chemicals were purchased from Merck (Darmstadt, Germany) and are either of analytical or HPLC grade.

## 3. Methods

### 3.1. DNA Manipulation

*C. sinensis genomic *DNA was extracted from the leaves obtained from Cameron Highlands, Malaysia, using standard methods [17]. The recombinant DNA techniques were carried out as described by Sambrook and Russell [18].

### 3.2. Construction of the Plasmids

**Figure 2:**
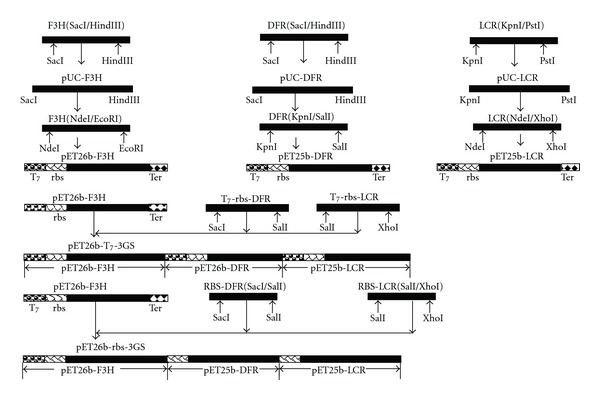
Diagrammatic presentation of the constructs.

Both plasmids (pET26b-T_7_-3GS and pET26-rbs-3GS) were constructed using the method proposed by Hwang et al. [14] and Chun et al. [19]. Using the purified genomic DNA as a template, appropriate primers (Table 1) were used to amplify the open reading frames of the coding regions of F3′H, DFR, and LCR resulting in 1107 bp F3H (SacI/HindIII), 1044 bp DFR (SacI/HindIII), and 1044 bp LCR (KpnI/PstI) fragments. The amplified fragments were cloned into the corresponding sites on pUC-18 to form pUC-F3H, pUC-DFR and pUC-LCR. The NdeI/EcoRI fragment derived from pUC-F3′H was cloned into pET26b between NdeI/EcoRI to form pET26b F3′H. Similarly, the KpnI/SalI and NdeI/XhoI fragments, which had been derived from pUC-DFR and pUC-LCR, respectively, were cloned into the corresponding sites of pET25b to form pET25b-DFR and pET25b-LCR. Using pET25b-DFR and pET25b-LCR as the templates, the SacI/SalI (containing T_7_-rbs-DFR and/or rbs-DFR) and SalI/XhoI (containing T_7_-rbs-LCR and/or rbs-LCR) fragments were, respectively, derived. In order to construct pET26b-T_7_-3GS or pET26-rbs-3GS, either T_7_-rbs-DFR and T_7_-rbs-LCR or rbs-DFR and rbs-LCR were introduced stepwise into pET26b-F3H (Figure 2).

### 3.3. Expression and Fermentation

Expression and fermentation conditions were carried out using the method proposed by [14]. *E. coli* BL21(DE3) harbouring pET26b-rbs-3GS and pET26b-PT_7_-3GS were precultured in Luria-Bertani liquid medium containing 5 *μ*g of kanamycin/mL at 30°C for 16 h with reciprocal shaking. Then, isopropyl *β*-D-thiogalactopyranoside (IPTG) was added at the concentration of 1 mM. After an incubation period of 2 h at 26°C, the culture was prepared in an appropriate volume of 1X SDS loading buffer and heated to 100°C for 3 min. The mixture was then loaded onto a sodium dodecyl sulphate (SDS) polyacrylamide gel to analyze for total proteins [18]. In order to identify the compounds produced by *E. coli* BL21 (DE3) harbouring pET26b-rbs-3GS and pET26b-PT7-3GS, a portion (2 mL) of the pre-culture was inoculated into 20 mL of M9 medium (containing 10–40 g/L glucose and 5 *μ*g of kanamycin/mL) and cultured for 5 h at 26°C in the presence of different concentrations of IPTG ranging from 0.25 mM to 1 mM. The cells were then harvested and a 50 mg (wet weight) portion of it was transferred into 20 mL of fresh M9 medium (containing 10–40 g/L glucose and 5 *μ*g of kanamycin/mL), IPTG (0.25 mM to 1 mM), eriodictyol (3 mM)) and cultivated at 26°C for 65 h with reciprocal shaking.

### 3.4. Extraction and Analysis of Catechins

The extraction of catechins was carried out using the method proposed by Hwang et al. [14]. The pH of culture broth was adjusted to pH 9.0 with 0.5 M NaOH and allowed to stand at room temperature for 1 h. The compounds in the broth were then extracted with 20 mL of ethyl acetate. The organic layer was evaporated at 40°C using rotary evaporator (Eyela N1001, Tokyo Rikakikai Co. Ltd), and the residue was dissolved in 30 *μ*L of methanol. The sample was analyzed using a high-performance liquid chromatography (HPLC) Model, Jasco CO-2065 Plus (Jasco Corporation, Japan) equipped with a reversed phase C-18 Purospher Star column, PDA detector with an acquisition wavelength set in the range of 200–400 nm and maintained at 40°C and a flow rate of 1 mL/min. The compounds were separated using a mobile phase with water-acetic acid (97 : 3) as solvent A and methanol (solvent B) by gradient elution. The mobile phase composition was 100% solvent A during the first minute and gradually decreased to 37% at 28 min. The mobile phase composition was then brought back to the initial conditions within 2 min for the next run [20]. The peaks were identified by comparing the retention times of the sample to those of standards.

## 4. Results

Recombinant *E. coli* BL(DE3), carrying the gene clusters (pET26b-T_7_-3GS and pET26-rbs-3GS) of the metabolite genes of *C. sinensis* produced two of the four major catechins, namely, (−)-epicatechin (0.01 mg/L) and (−)-epicatechin gallate (0.36 mg/L), in addition to (±)-catechin hydrate (0.13 mg/L) and (−)-catechin gallate (0.04 mg/L) in alkaline conditions (pH 9.0), after 1 h at room temperature and when cultivated at 26°C in M9 medium consisting of 40 g/L glucose, 1 mM IPTG, and 3 mM eriodictyol, after 65 h of incubation with reciprocal shaking (Figure 4(a)). However, those cultures which were not supplemented with eriodictyol did not produce catechins.

The results from SDS-PAGE produced bands of corresponding theoretical sizes of the expected functional proteins, namely, CsF3H (41.46 kDa, 368aa), CsDFR (38.69 kDa, 347aa), and CsLCR (37.23 kDa, 347aa), which were similar to previously published results [21, 22]. These bands were excised and identified through Peptide sequencing by mass spectrometry.

**Figure 3:**
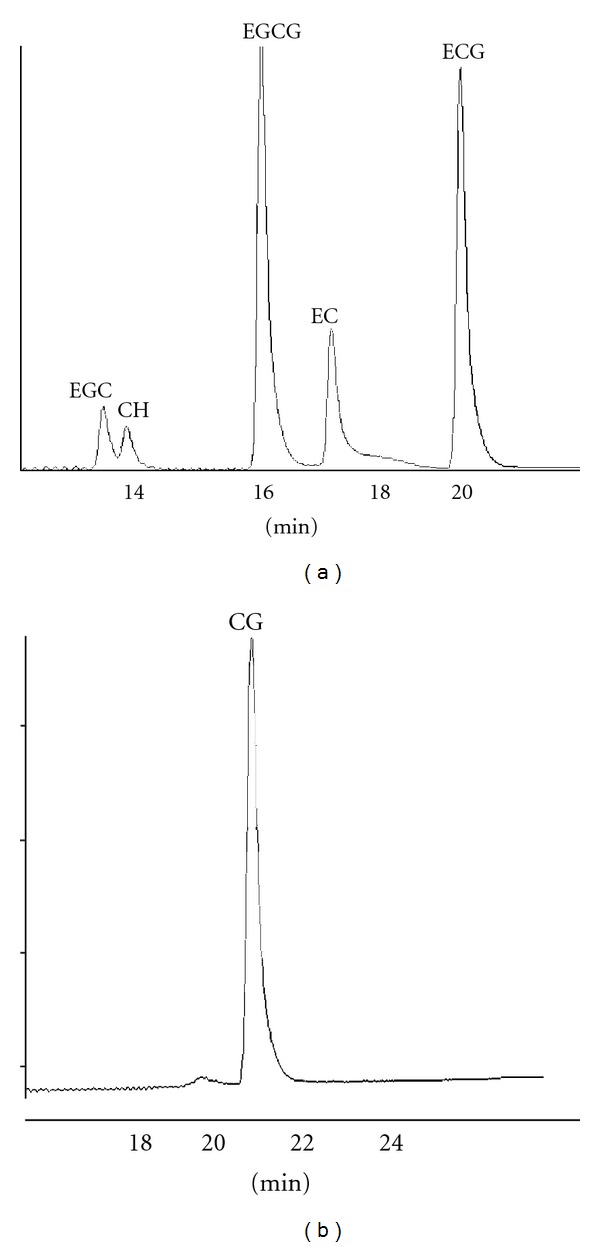
(a) HPLC chromatograms of standard catechins (EC: (−)-epicatechin; ECG: (−)-epicatechin gallate; EGC: epigallocatechin; EGCG: epigallocatechin gallate; CH: (+)-catechin Hydrate); (b) chromatogram of (−)-catechin gallate standard.

**Figure 4:**
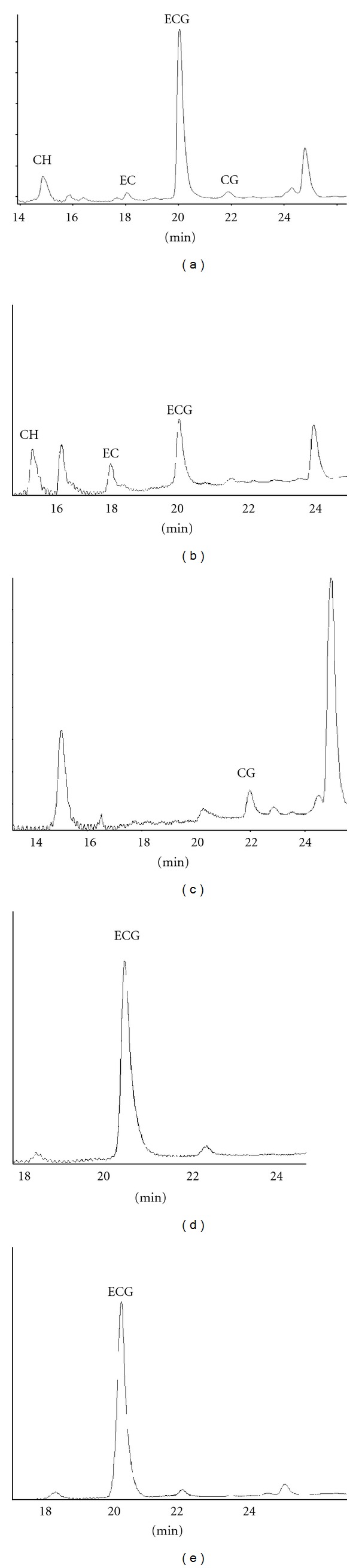
(a) Chromatogram of (±)-catechin hydrate (0.13 mg/L), (−)-epicatechin (0.01 mg/L), (−)-epicatechin gallate (0.36 mg/L), and (−)-catechin gallate (0.04 mg/L) in M9 medium supplemented with 1 mM IPTG and 40 g/L glucose and 3 mM eriodictyol; (b) chromatogram of (±)-catechin hydrate (0.04 mg/L), (−)-epicatechin (0.02 mg/L), (−)-epicatechin gallate (0.04 mg/L) in M9 medium supplemented with 1mM IPTG and 10 g/L glucose and 3 mM eriodictyol; (c) chromatogram of (−)-catechin gallate (0.05 mg/L) and unknown compounds in cultures without IPTG; (d) chromatogram of biosynthesized (−)-epicatechin gallate, (0.08 mg/L) in M9 medium supplemented with 0.5 mM IPTG and 1 mM eriodictyol; (e) chromatogram of (−)-epicatechin gallate (0.34 mg/L) production at 1 mM IPTG and 3 mM eriodictyol in M9 medium.

The retention times of catechin standards used in this study are shown in Figure 3 and were similar to the retention times of the compounds produced during this study. The retention times were 14.86, 17.68, 20.04, and 21.88 min for (+)-catechin hydrate, (−)-epicatechin, (−)-epicatechin gallate, and (−)-catechin gallate, respectively (Figure 4(a)). In addition, there were four other unidentified compounds with retention times of 15.91, 18.02, 24.65, and 24.79 min.

To determine the impacts of increased glucose concentration, the cultures were cultivated in 3 mM eriodictyol and graded concentrations of glucose (10–40 g/L) in M9 minimal salts. Results showed an increase in the concentration of (±)-catechin hydrate; from 0.04 to 0.13 mg/L, (−)-epicatechin gallate; 0.04 to 0.36 mg/L and (−)-catechin gallate; 0 to 0.04 mg/L with increase in glucose concentration from 10 to 40 g/L. However, the concentration of (−)-epicatechin was found to decrease (0.02 to 0.01 mg/L) with increase in glucose concentration (Figures 4(a) and 4(b)).

## 5. Discussion

This study demonstrates the production of (−)-epicatechin, (−)-epicatechin gallate, (±)-catechin hydrate, and (−)-catechin gallate using the recombinant strains of *E. coli *BL21 (DE3) cells carrying the metabolite genes of *C. sinensis. *Meanwhile, several prior studies have shown *C. sinensis* as one of the major sources of catechins [23]. Additionally, the analysis of the genes has made defined mRNA sequences, including coding regions and open reading frames available for references [21, 22]. Furthermore, the expression of these genes, in relation to the concentration of catechins, has been demonstrated as well [3, 24]. In the present study, the proposed catechin biosynthetic pathway was adopted [1]. The successful production of these compounds has validated the proposed pathway for catechin biosynthesis.

In line with the metabolic engineering approach, the biosynthetic pathway was introduced into *E. coli *BL21 (DE3) on a pET vector. It is crucial to note that *E. coli *BL21 (DE3) lacks ompT (i.e., the outer membrane protease that degrades protein during purification) [25]. Therefore, to ensure a selective and an active transcription of the genes of interest, the pET vector which is controlled by a bacteriophage T_7_ promoter induced by IPTG and compatible to the host was selected for use in the current study. Meanwhile, perturbations were introduced in the form of a number of promoters and ribosome-binding sequences. In addition, the source of genes was from a homologous origin in contrast to the previous studies which were comprised of heterologous clusters [12] to enhance the production the major catechins found in *C. sinensis.* Contrary to the expectation of producing all the major catechins, only two, namely, (−)-epicatechin, (−)-epicatechin gallate, were produced in addition to (±)-catechin hydrate and (−)-catechin gallate.

Studies in enzymology have indicated the suboptimum pH for the activity of catechin biosynthetic enzymes to be 7.5 [26]. To enhance the activity of expressed gene products in this study, the pH values of the cultures were changed from an initial acidic condition to high alkanine conditions (pH 9.0) and allowed to stand for 1 hour. The decrease in the production of (−)-epicatechin is accompanied by an increase in the amount of other catechins and the reason for this trend is poorly understood.

The production of catechins by the recombinant strains was possible in the absence of IPTG (Figure 4(c)). Moreover, there was a similarity in the retention times of the unidentified compounds with that of IPTG-induced cultures. The reason may be due to the basal expression of T_7_ RNA polymerase gene in BL21 (DE3). As eriodictyol and IPTG are expensive ingredients, the feasibility of using these compounds for industrial production is therefore very low. Nevertheless, the production of (−)-epicatechin gallate (0.08 mg/L) was possible at lower concentrations of 0.5 mM IPTG and 1 mM eriodictyol (Figure 4(d)). Hence, the choice of eriodictyol in this study was based on the substrate specificity for the enzyme activity in flavonoid biosynthesis, as previously reported [26].

As reported previously, supplementation with glucose leads to a reduction in the basal expression of lacUV5 promoter in the T_7_ polymerase-based expression systems through its influence on the level of cyclic adenosine monophosphate (cAMP). It has also been suggested that cAMP/CAP activator complexes increase the production of T_7_ polymerase and thus initiate transcription in the long run [27, 28]. Apparently, more of the different catechins were produced at higher glucose concentrations (Figure 4(a)) than that at the minimal levels (Figure 4(e)). This can be attributed to increased recombinant plasmid stability coupled with high target protein yield with increase in glucose concentration which is in agreement with previous reports [29].

The high contents of the various catechins obtained in this study are in agreement with the previous enzymology studies carried out on flavonoid biosynthesis in *C. sinensis *[26]. Apart from that, the presence of ribosome binding sites (RBS), which facilitate the binding of mRNA and protein translation [30], aids in the high expression of the three genes. Moreover, the production of (−)-epicatechin and (−)-epicatechin gallate is indication of the similarities of the enzymatic biosynthetic pathways of the two compounds with (±)-catechin hydrate and (−)-catechin gallate.

Thus, future research should focus on the isolation of the identified compounds, as well as their potency in relation to the previously reported functionalities. As for the unidentified compounds, NMR will be used to determine their structure and to find out whether or not they are newly engineered compounds. Since the feasibility of using eriodictyol and IPTG for industrial production is not economical at all, other genes will be cloned in the pathway and arranged in a cluster to produce the precursors. The possibility of using heterologous sources to produce major catechins will also be explored.

## Figures and Tables

**Table 1:** Primers used for the study.

Primer Name	Sequences
F3H__(SacI)_-F	**GCG GAG CTC** GAG ATG GCG CCA ACA ACA
F3H__(HindIII)_-R	**GAT AAG CTT** ACA CTC TCA AGC AAA AAT CTC
DFR__(SacI)_-F	**GCG GAG CTC **ATC ATG AAA GAC TCT GTT
DFR__(HindIII)_-R	**GAT AAG CTT** TTA AAC CTT GTT GCC ATT
LCR__(KpnI)_-F	**GTA GGT ACC** GCC ATG GCA ATG GCA ATG
LCR__(PstI)_-R	**GCG CTG CAG** GTG CCT TCA GTT CTG CAA
F3H__(NdeI)_-F	**GCG CAT ATG** GAG ATG GCG CCA ACA ACA
F3H__(EcoRI)_-R	**GAT GAA TTC** ACA CTC TCA AGC AAA AAT CTC
DFR__(KpnI)_-F	**GCG CCA TGG** ATC ATG AAA GAC TCT GTT
DFR__(SalI)_-R	**GAT GTC GAC** TTA AAC CTT GTT GCC ATT
LCR__(NdeI)_-F	**GTA CAT ATG** GCC ATG GCA ATG GCA ATG
LCR__(XhoI)_-R	**GCG CTC GAG** GTG CCT TCA GTT CTG CAA
_SacI-T_7_-rbs_-DFR-F	GCG** GAG CTC** CGA TCC CGC GAA ATT AAT
_SacI-T_7_-rbs_-DFR-R	GAT** GTC GAC** TTA AAC CTT GTT GCC ATT
_ SacI-rbs_-DFR-F	AAC **GAG CTC** AAG AAG GAG TAT ACA TAT
_ SalI-rbs_-DFR-R	GAT **GTC GAC** TTA AAC CTT GTT GCC ATT
_SacI-T_7_-rbs_-LCR-F	TCA **GTC CGA** TCC CGC GAA ATT AAT
_Xhol-T_7_-rbs_-LCR-R	GCG **CTC GAG** GTG CCT TCA GTT CTG CAA
_ SalI-rbs_-LCR-F	AAC **GTC GAC** AAG AAG GAG TAT ACA TAT
_ XhoI-rbs_-LCR-R	GCG **CTC GAG** GTG CCT TCA GTT CTG CAA

*Sequences in bold are restriction enzyme sites of degenerate primers.
